# Demographic characteristics influencing the stem subsidence in total hip arthroplasty: an imaging study

**DOI:** 10.1007/s00402-023-05054-y

**Published:** 2023-09-29

**Authors:** Filippo Migliorini, Nicola Maffulli, Marco Pilone, Erlis Velaj, Ulf Krister Hofmann, Andreas Bell

**Affiliations:** 1 Department of Orthopaedics, Trauma, and Reconstructive Surgery, University Clinic Aachen, RWTH Aachen University Medical Centre, Pauwelsstraße 30, 52064 Aachen, Germany; 2 Department of Orthopaedics and Trauma Surgery, Academic Hospital of Bolzano (SABES-ASDAA), Teaching Hospital of Paracelsus Medical University (PMU), 39100 Bolzano, Italy; 3 Department of Orthopaedic and Trauma Surgery, Hospital Sant’Andrea, University of Rome La Sapienza, Rome, Italy; 4 School of Pharmacy and Bioengineering, Keele University School of Medicine, Thornburrow Drive, Stoke On Trent, England; 5 Barts and the London School of Medicine and Dentistry, Centre for Sports and Exercise Medicine, Mile End Hospital, Queen Mary University of London, 275 Bancroft Road, London, E1 4DG England; 6 Residency Program in Orthopedics and Traumatology, University of Milan, Milan, Italy; 7 Department of Orthopedics, Eifelklinik St. Brigida, 52152 Simmerath, Germany

**Keywords:** Hip, Arthroplasty, Implant, Subsidence

## Abstract

**Introduction:**

The present study evaluated whether patient demographic characteristics influence the subsidence of the stem in total hip arthroplasty (THA). The following characteristics were evaluated: age, height, weight, and sex. The association between the time elapsed from the THA implantation and the amount of stem subsidence was also investigated.

**Methods:**

The records of patients who underwent THA in the period between 2016 and 2023 were accessed. All patients underwent two-staged bilateral THA using cementless DePuy collarless Corail (DePuy Synthes, Raynham, MA, USA) stems. The following parameters were measured and compared to assess stem subsidence: distance from the proximal femur at the stem bone interface and the medial apex of the regular triangle built within the trochanter minor (point A); distance from the medial apex of the regular triangle built within the trochanter minor and the distal portion of the femoral stem (point B).

**Results:**

Overall, 294 patients were included. 62% (182 of 294 patients) were women. 45% (134 of 296 THAs) were on the right side. The mean age was 64.9 ± 10.4 years. The mean BMI was 28.3 ± 5.1 kg/m^2^. The mean length of the follow-up was 14.4 ± 11.0 months. The mean subsidence in point A was 2.1 mm (*P* < 0.0001), and that in point B was 3.1 mm (*P* < 0.0001). There was evidence of a weak positive association between patient weight (*P* < 0.0001), age (*P* = 0.03), follow-up (*P* = 0.002) and the amount of stem subsidence. Patient height did not demonstrate any association with the amount of stem subsidence (*P* = 0.07). There was no difference in stem subsidence between women and men (*P* = 0.9).

**Conclusion:**

Stem subsidence in THA using cementless DePuy collarless Corail implants is approximately 2.6 mm after 14.4 months. Greater patient weight, age, and longer time elapsed from THA implantation were associated with greater stem subsidence. Patient height and sex did not demonstrate any influence on the amount of stem subsidence. These results must be considered in light of the limitations of the present study.

**Supplementary Information:**

The online version contains supplementary material available at 10.1007/s00402-023-05054-y.

## Introduction

Osteoarthritis (OA) of the hip is a common cause of pain and disability [1]. In selected patients, total hip arthroplasty (THA) improves functional outcomes, favorably impacting patient quality of life and participation in recreational activities [2]. To guarantee survivorship of THA, a stable structural and functional connection between the underlying bone and the implant is necessary. However, the current evidence demonstrated that the femoral component of THA, although well integrated with the surrounding bone, can undergo minimal displacement. This process of “distalisation” of the stem in absence of implant loosening is called subsidence [3]. Most radiographic subsidence is observed within six to eight weeks postoperatively and is more evident in cementless implants [4]. Subsidence up to 3 mm is considered acceptable, whereas a stem migration greater than 5 mm could lead to implant failure [5]. Femoral stem design may influence stem subsidence [6].

Despite the increasing number of clinical investigations, the influence of patient characteristics on stem subsidence in THA is still debated. The present study assesses stem subsidence using the anteroposterior radiographs of the pelvis of patients who underwent two-staged bilateral THA. The purpose of the present study is to assess the amount of stem subsidence following primary THA and to evaluate whether patient demographic characteristics influence its subsidence.

## Methods

### Study design

The present retrospective study was performed according to the Strengthening the Reporting of Observational Studies in Epidemiology (STROBE) [7]. The present study was a retrospective analysis of prospectively collected data. The databases of the Department of Orthopaedic Surgery of the Eifelklinik St. Brigida, Simmerath, Germany and the Department of Orthopaedic, Trauma, and Reconstructive Surgery of the University Hospital RWTH Aachen, Germany, were accessed. Data from all operated patients are collected prospectively following the standard highlighted in the “Endocert” (EndoCert certificate, Centres of German Endoprosthetic, German Society for Orthopedics and Traumatology), which supervises and certifies the quality of the arthroplasties. In December 2022, the records of patients who underwent THA between 2016 and 2022 were accessed. All patients who underwent two-staged bilateral THA at our institutions were retrieved. The present study was approved by the Ethics Committee of the RWTH University of Aachen (project ID: EK128/19) and conducted according to the principles expressed in the Declaration of Helsinki.

### Eligibility criteria

The inclusion criteria were: (1) symptomatic OA secondary to dysplasia, or femoral head necrosis, and idiopathic; (2) symptomatic OA grade II to IV according to the Kellgren–Lawrence classification [8]; (3) completion of postoperative antithrombotic prophylaxis; (4) completion of postoperative prophylaxis for heterotopic ossification; (5) a minimum of six months between the implantation of the ipsi- and the contralateral THA; (6) patients who underwent postoperative inpatient rehabilitation; (7) patients being able to understand the nature of the treatment. The exclusion criteria were: (1) hip OA secondary to trauma; (2) neoplastic diseases; (3) pregnancy; (4) any blood abnormalities; (5) severe peripheral neuropathy, vascular diseases, or presence of peripheral ulcers; (6) osteoporosis which requires component cementation; (7) concomitant intake of anticoagulants or calcitonin; (8) patients who underwent revision surgery for any reason; (9) patients who had any complication which may have impaired weight-bearing or gait; (10) other omitted criteria which may have influenced the results of the present investigation.

### Surgical technique

All patients received a 1.5 g single shot of intravenous cefuroxime. All surgeries were performed by six senior surgeons using the Watson–Jones anterolateral approach [9]. The implant used for THA was the cementless DePuy (DePuy Synthes, Raynham, MA, USA) collarless Corail stem and a Pinnacle acetabular cup, an oxinium or ceramic femoral head, and a high-density crosslinked polyethylene (XLPE) inlay. Anti-thrombotic prophylaxis with Rivaroxaban, 10 mg daily for 6 weeks, started 12 h after the index procedure was implemented. Three weeks of ibuprofen 600 mg thrice daily as prophylaxis for heterotopic ossification was administered. Patients were followed by a team of physiotherapists during hospitalization. Quadriceps strength exercise started on the first postoperative day. On the same day, patients mobilized weight-bearing as tolerated using a forearm support frame. By the third postoperative day, patients usually progressed to mobilization using crutches. An inpatient physiotherapy program in external rehabilitation institutes was set for every patient.

### Outcomes of interest

**Fig. 1:**
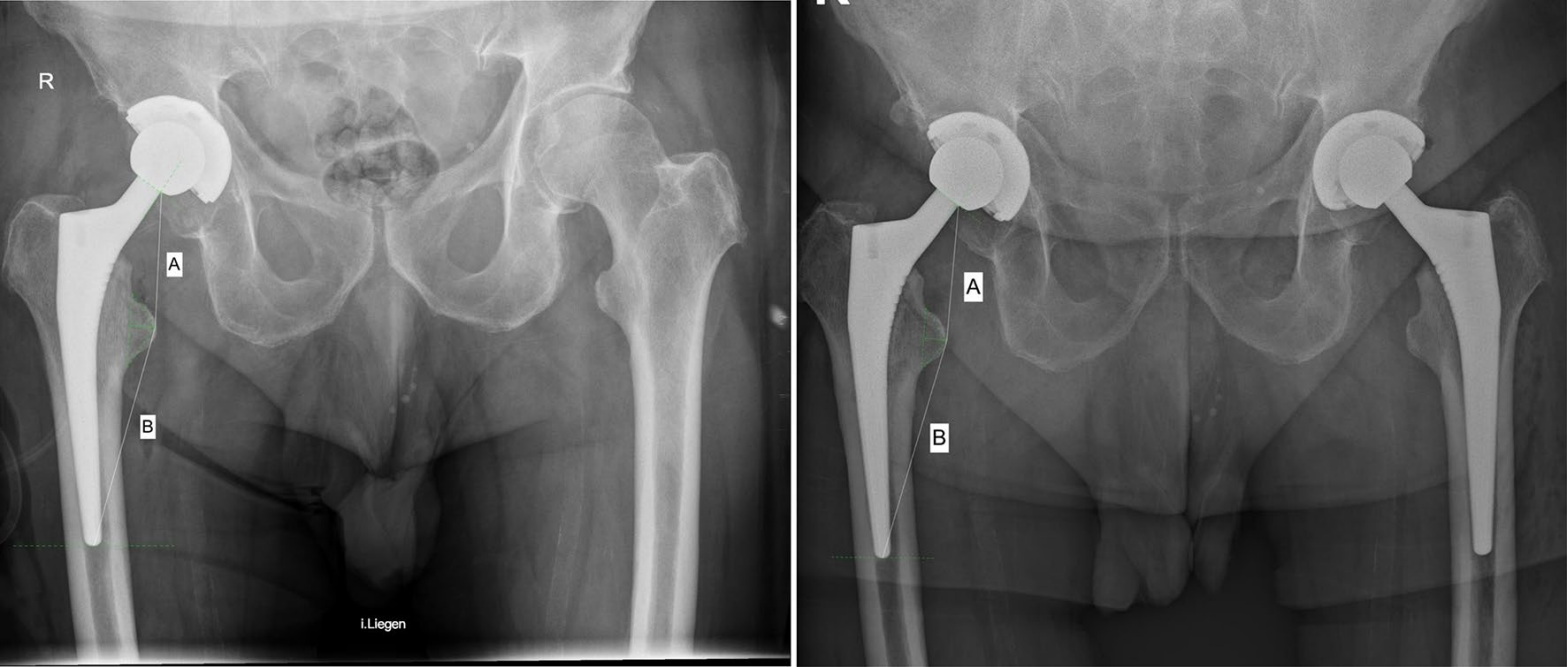
Evidence of subsidence of 1.0 mm after 7 months of follow-up in a male patient of 72 years old on anteroposterior radiographs of the pelvis (**A**: following the first THA; **B**: following the implantation of the contralateral THA)

All patients who underwent two-staged bilateral THA were considered in the present investigation. Data concerning the date of surgery, age, sex, weight, and height of the patients were collected. On admission, patients underwent anteroposterior radiography of the pelvis. The anteroposterior radiographs taken following the first THA were compared with those of the same side taken at the time of the contralateral THA implantation (Fig. 1). In doing so, patients avoided additional radiographs for research purposes.

**Fig. 2:**
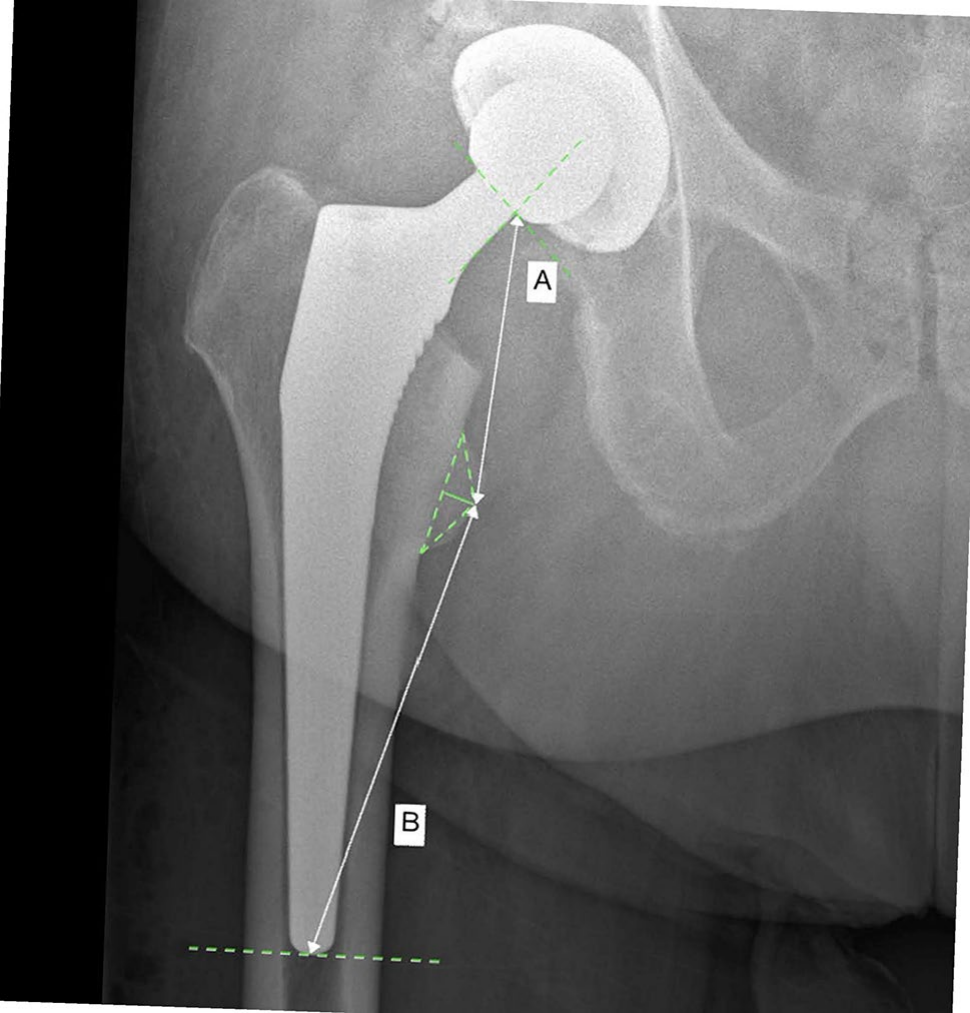
Reference parameters (**A**: distance from the proximal femur at the stem bone interface and the medial apex of the triangle draw within the superior, inferior and medial margins of the lesser trochanter; **B**: distance from the medial apex of the regular triangle drawn within the superior, inferior and medial margins of the lesser trochanter and the distal portion of the femoral stem)

The amount of subsidence was assessed by a blinded assessor who was not involved in the clinical management of the patients. The imaging references used to assess stem subsidence are shown in Fig. 2.

### Statistical analysis

Statistical analyses were performed by the main author (F.M.) using the IBM SPSS software (version 25). For descriptive statistics, mean and standard deviation were evaluated. To evaluate subsidence in women and men, the mean difference (MD) effect measure was calculated. The two tailed unpaired T-test was performed, with values of *P* < 0.05 considered statistically significant. A multiple linear model regression diagnostic was conducted using STATA/MP 16.1 (StataCorp, College Station, TX) to investigate whether the time elapsed from THA and age, height, and weight of the patients exerted an influence on stem subsidence. The string was set to exclude extreme outliers. For pairwise correlation, the Pearson Product-Moment Correlation Coefficient (*r*), with values of + 1 and -1 indicating a positive and a negative linear correlation, respectively. Values of 0.1 <| *r* |< 0.3, 0.3 <| *r* |< 0.5, and | *r* |> 0.5 were considered to have small, medium, and strong correlation, respectively. The test of overall significance was performed through the χ^2^ test, with values of P < 0.05 considered statistically significant. Scatter plots were also performed and added as supplementary material.

## Results

### Recruitment process

**Fig. 3:**
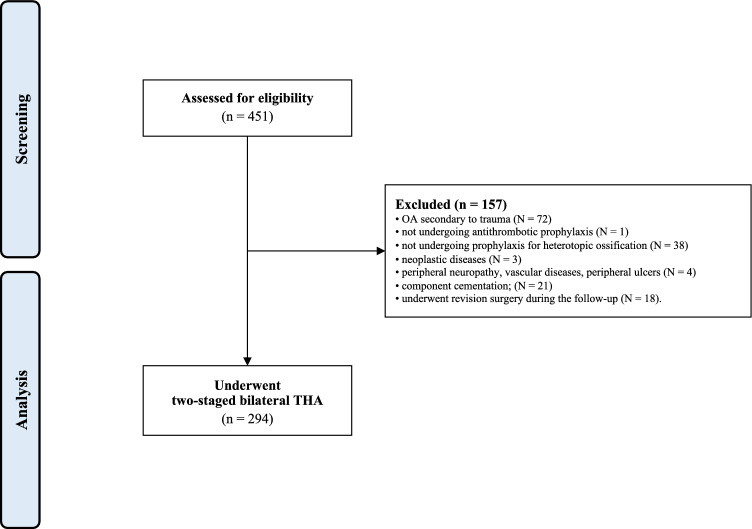
Diagram of the recruitment process

Data from 451 procedures were retrieved. Of them, 157 were not considered in the present study due to the following reasons: OA secondary to trauma (*N* = 72), not undergoing antithrombotic prophylaxis (*N* = 1), not undergoing prophylaxis for heterotopic ossification (*N* = 38), neoplastic diseases (*N* = 3), severe peripheral neuropathy, vascular diseases, or presence of peripheral ulcers (*N* = 4), component cementation (*N* = 21), underwent revision surgery during the follow-up (*N* = 18). Finally, 294 were identified and included in the present analysis (Fig. 3).

### Patient demographics

Overall, 294 patients were included. 62% (182 of 294 patients) were women. 45% (134 of 296 THAs) were implanted on the right side. The mean age was 64.9 ± 10.4 years. The mean BMI was 28.3 ± 5.1 kg/m^2^. The mean length of the follow-up was 14.4 ± 11.0 months. Demographic data are shown in Table 1.

**Table 1:** Demographic data of the patients (FU: follow-up)

Endpoint	Value
Side (*right*)	62% (182 of 294)
Women	45% (134 of 296)
Height (*cm*)	170.4 ± 9.5
Weight (*kg*)	82.5 ± 17.1
BMI (*kg/m*^*2*^)	28.3 ± 5.1
Age (*years*)	64.9 ± 10.4
FU (*months*)	14.4 ± 11.0

### Outcomes of interest

The overall cementless collarless stem subsidence following THA was 2.6 ± 4.2 mm (*P* < 0.0001) at 14.4 ± 11.0 months. Table 2 reports the amount of subsidence in zone A and B.

**Table 2:** Main results

Endpoint	At Baseline	At last FU	MD	T	Effect size	95% CI	*P*
C	58.3 ± 7.8 (75.7 to 30.2)	56.2 ± 8.1 (73.1 to 30.2)	2.1	11.001	0.64	2.4542, 1.7098	< 0.0001
D	106.8 ± 15.0 (134.2 to 59.2)	109.9 ± 15.3 (138.5 to 61.1)	3.1	11.186	0.65	2.531, 3.6118	< 0.0001

### Pairwise correlations

There was evidence of a weak positive association between patient weight (*r* = 0.2; *P* < 0.0001, Table 3), age (*r* = 0.1; *P* = 0.03, Table 3), the time elapsed from surgery (*r* = 0.2; *P* = 0.002, Table 3), and the amount of stem subsidence (Table 3). Patient height did not demonstrate any association with the amount of stem subsidence (*P* = 0.07, Table 3).

**Table 3:** Results of the pairwise correlations

Endpoints	*r*	*P*	
Height	0.15	0.07	
Weight	0.22	< 0.0001	
Age	0.13	0.03	
Time span (*months*)	0.18	0.002	

### Subgroup analysis: women vs men

There was no difference in stem subsidence between women and men (*P* = 0.9, Table 4).

**Table 4:** Comparison of stem subsidence between women and men

Endpoint	Men	Women	MD	Effect size	*T*	*P*
Overall	2.5 ± 3.0	1.8 ± 3.4	0.7	0.21	1.71	0.9

## Discussion

According to the main findings of the present study, cementless DePuy collarless Corail stem subsidence in THA is approximately 2.6 mm by 14 months from the implantation. Greater patient weight, age, and longer time elapsed from THA implantation were associated with greater stem subsidence. Patient height and sex did not demonstrate any influence on the amount of stem subsidence.

The final stability of the implant is crucial to avoid aseptic loosening and it is reached in two steps: primary and secondary stability [10]. Primary stability depends on the micro-movement of the stem immediately after surgery [11]. The early subsidence in cementless collarless stems can be related to improper anchoring during implantation rather than a true loosening of the implant [4]. This may be due to an insufficient impaction of the implant into the cancellous bone during the surgery [12]. The body weight generates a force from the implant onto the bone causing further compression of the implant, resulting in subsidence until mechanical stability is obtained [5]. Secondary stability is guaranteed by osseointegration that allows proper anchorage of the implant to the surrounding bone [13]. Osteointegration follows three phases: incorporation of implants by woven bone formation, deposition of lamellar bone fibers, as the response of bone mass to loads, and final bone remodeling [14]. Osteointegration is influenced by many factors: biocompatibility of the implant, positioning of the implant, biomechanics of the implant, loading conditions, surgical technique, and healing phase [14–16]. Streit et al. [17] analyzed subsidence in 158 cementless THAs. After 21 years of follow-up, they found a statistically significant association between early subsidence and aseptic loosening [17]. The present study showed a weak association between body weight and subsidence. This result is in accordance with Stihsen et al.'s [18] study. 102 patients were analyzed, and subsidence was greater in patients weighting 75 kg or more than in lighter patients. Interestingly, BMI was not associated with stem migration. In a previous study, though not statistically significant, a greater tendency of subsidence in obese patients (BMI > 30 kg / m^2^) was observed compared to a non-obese control group [19]. Leiss et al. [12] analyzed the influence of partial and full weight-bearing on subsidence. Patients with a full weight-bearing rehabilitation protocol showed greater stem migration than did the control group. Physiological loads allow the implant to reach the proper position and help osseointegration of the implant [20]. Body weight helps the stem to reach its final and physiological position in the medullary canal, with full cortical contact [12].

No association was present between gender and stem migration. This result is confirmed by other studies [21, 22]. An interesting explanation of these results is the role of bone mineral density (BMD) in stem migration. BMD decreases in post-menopausal females [23]. An RCT study on 62 patients (mean age 64) showed no association between BMD and subsidence [24]. The role of gender as a risk factor for subsidence is still controversial. Johanson et al. [25] showed greater stem migration in males, suggesting that these results could have been influenced by the different morphology of the femoral medullary canal.

Another factor that influences BMD is age [26]. Many studies suggest that cementation of THA after 75 years old may reduce of the revision rate [27–29]. Moritz et al. [30] analyzed the association of subsidence with the quality of inter-trochanter cancellous bone in 61 female patients, showing an association between age and BMD, but BMD did not influence stem migration. The present study showed a statistically significant association between subsidence and age. This result is confirmed also in a previous study which found greater subsidence in elderly patients [31]. However, another study on collarless Corail stem showed greater subsidence in the younger patients [5]. The authors suggested that this result can be explained by the highest activity level of these patients, even if the activity level was not evaluated in their study.

In our study, migration of the stem was associated with time elapsed from surgery. The short–mid-term follow-up might have influenced this result. Although the subsidence is maximal in the first 3 months and tends to stabilize by 24 months, little stem migration is still present over the years [32, 33]. For this reason, we decided to include only patients with a minimum of 6 months of follow-up. Critchley et al. [34] described stem migration patterns in a 14-year follow-up study on 29 patients. The mean subsidence after 6 years was 0.62 mm and the mean subsidence after 14 years was 0.70 mm. There was no statistically significant difference between subsidence after 6 years and after 14 years [35]. Stem migration reaches a plateau after the first 2 years, when the stem is strongly osseointegrated and minimal variation in subsidence has to be considered as a result of physiological bone remodeling [35]. Similarly, Aro et al. [36] reported no stem subsidence between 2 and 9 years after surgery. Streit et al. [17] did not find any statistically significant association between aseptic loosening and stem subsidence after the first 2 years.

The present study showed an overall subsidence of 2.6 mm. A previous study which investigated the subsidence of cement-free Corail stems showed similar results at 24 months (subsidence 2.2 mm). Ries et al. in a study on 231 patients also operated with Corail stems, measured a mean subsidence of 2.9 mm after 7 months of follow-up [37]. The implantation of the Corail stem requires a high level of press fit to achieve the correct implantation and avoid aseptic loosening [38].

Our study presented some limitations. The follow-up period is relatively short and a longer follow-up could investigate whether subsidence might influence the rate of aseptic loosening. The presence of patients who undergo contralateral THA may increase the selection bias of the present study. Patients who were not completely satisfied with the outcome might have accepted to undergo further surgery on the contralateral side. This could have underestimated the real amount of stem subsidence. Only axial stem migration was analyzed in our patients, and rotational and tilting subsidence was not taken into account.

Software and online platforms developed to assess subsidence are available. In this instance, “Ein Bild Roentgen Analyze—femoral component analysis” (EBRA-FCA) is an accurate tool to assess stem migration [39]. However, this software is not commonly used in clinical practice. Therefore, we reported data from a simple method to assess subsidence. Despite the present methodology might not be as accurate as dedicated software, it is simple and fast to use. The stem design might have an impact on subsidence [40]. The primary stability of the stem depends also on implant morphology and surgery [41]. Indeed, cemented stem migrates less than the uncemented stem [24, 42] and the collared Corail femoral stem migrates less than the uncollared Corail femoral stem [5]. Finally, bone mineral density (BMD) was not evaluated in the present study. The influence of BMD on stem subsidence is still controversial [24, 43, 44], and additional studies using high-resolution imaging modalities of local bone quality are required. Following the principles highlighted in the Endocert, at our institutions, preoperative two-dimensional planning of the component type, brand, and position was made. However, whether incongruities between the preoperative surgical planning and the final position and size of the stem may have influenced the amount of subsidence has not been investigated in the present study. We did not include patients who underwent revision THA as additional factors may have influenced the amount of subsidence and could have led to a bias in the standardization of procedures and results. This limitation should be overcome by future investigations including patients with aseptic loosening of the femoral component.

## Conclusion

According to the main findings of the present study, cementless DePuy collarless Corail stem subsidence in THA is approximately 2.6 mm after 14 months of follow-up. Greater patient weight, age, and longer time elapsed from THA implantation were associated with greater subsidence. On the other hand, patient height and sex did not demonstrate any influence on the amount of stem subsidence. These results must be considered in light of the limitations of the present study.

### Supplementary Information

Below is the link to the electronic supplementary material.

Supplementary file1 (PDF 136 kb)

## Data Availability

Data is available at reasonable request to the main author (migliorini.md@gmail.it).
