# Supplementary figures and images for: Demographic characteristics influencing the stem subsidence in total hip arthroplasty: an imaging study
Source: Arch Orthop Trauma Surg. 2023 Sep 29;144(2):887–94. doi: 10.1007/s00402-023-05054-y (PMC10822810; doi:10.1007/s00402-023-05054-y)

Age

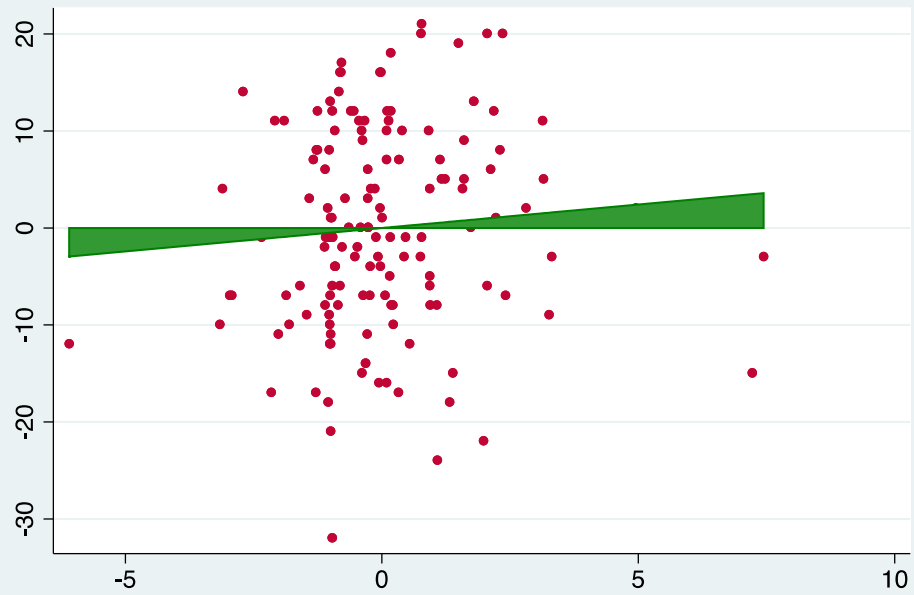

Height

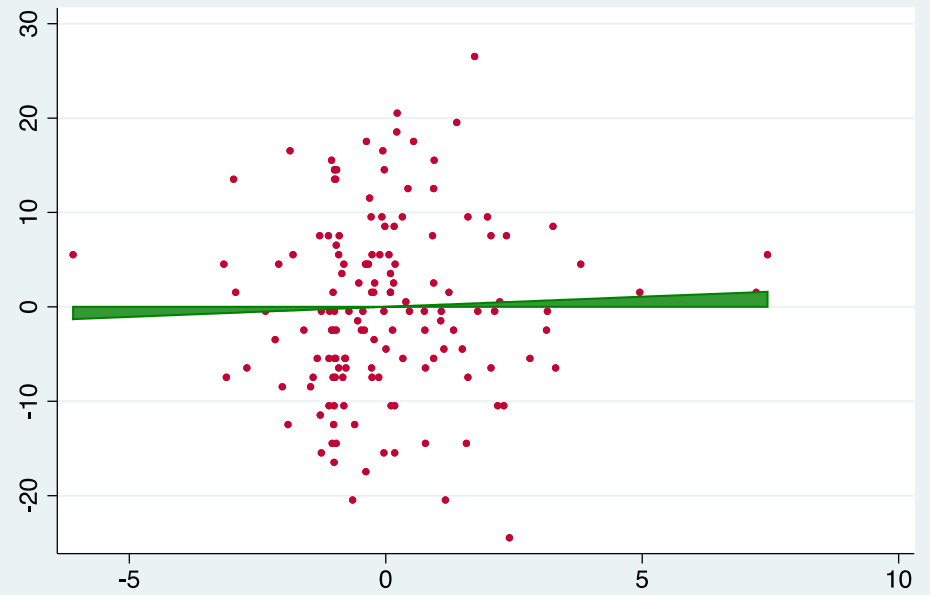

Time span

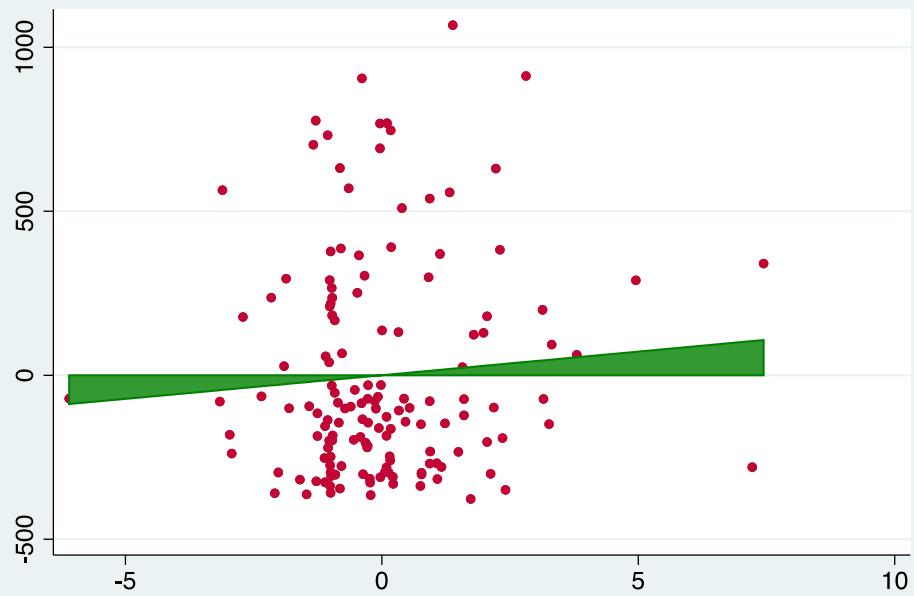

Weight

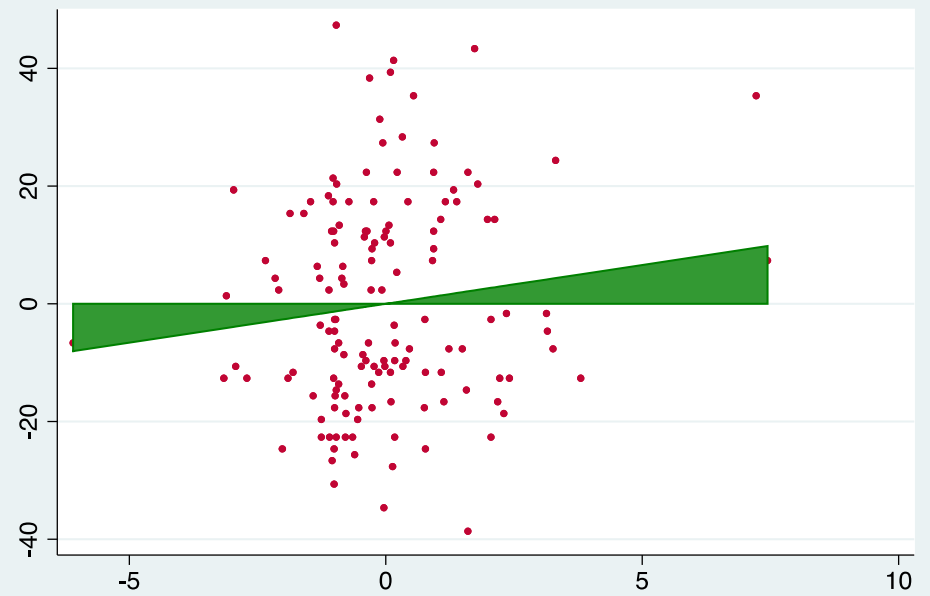

Supplement: Supplementary file 1 — Supplementary file1 (PDF 136 kb) [file 402_2023_5054_MOESM1_ESM.pdf]
